# Concordance of Epileptic Networks Associated with Epileptic Spikes Measured by High-Density EEG and Fast fMRI

**DOI:** 10.1371/journal.pone.0140537

**Published:** 2015-10-23

**Authors:** Vera Jäger, Matthias Dümpelmann, Pierre LeVan, Georgia Ramantani, Irina Mader, Andreas Schulze-Bonhage, Julia Jacobs

**Affiliations:** 1 Department of Neuropediatrics and Muscular Diseases, University Medical Center Freiburg, Freiburg, Germany; 2 Section for Epileptology, University Medical Center Freiburg, Freiburg, Germany; 3 Medical Physics, University Medical Center Freiburg, Freiburg, Germany; 4 Department for Neuroradiology, University Medical Center Freiburg, Freiburg, Germany; University of Electronic Science and Technology of China, CHINA

## Abstract

**Objective:**

The present study aims to investigate whether a newly developed fast fMRI called MREG (magnetic resonance encephalography) measures metabolic changes related to interictal epileptic discharges (IED). For this purpose BOLD changes are correlated with the IED distribution and variability.

**Methods:**

Patients with focal epilepsy underwent EEG-MREG using a 64 channel cap. IED voltage maps were generated using 32 and 64 channels and compared regarding their correspondence to the BOLD response. The extents of IEDs (defined as number of channels with >50% of maximum IED negativity) were correlated with the extents of positive and negative BOLD responses. Differences in inter-spike variability were investigated between interictal epileptic discharges (IED) sets with and without concordant positive or negative BOLD responses.

**Results:**

17 patients showed 32 separate IED types. In 50% of IED types the BOLD changes could be confirmed by another independent imaging method. The IED extent significantly correlated with the positive BOLD extent (p = 0.04). In 6 patients the 64-channel EEG voltage maps better reflected the positive or negative BOLD response than the 32-channel EEG; in all others no difference was seen. Inter-spike variability was significantly lower in IED sets with than without concordant positive or negative BOLD responses (with p = 0.04).

**Significance:**

Higher density EEG and fast fMRI seem to improve the value of EEG-fMRI in epilepsy. The correlation of positive BOLD and IED extent could suggest that widespread BOLD responses reflect the IED network. Inter-spike variability influences the likelihood to find IED concordant positive or negative BOLD responses, which is why single IED analysis may be promising.

## Introduction

Patients with focal refractory epilepsy profit from identifying brain regions which generate epileptic spikes and seizures. A better understanding of this epileptic focus and associated spike networks will allow to identify candidates for epilepsy surgery and other treatments such as intracranial stimulation [1,2]. For presurgical evaluation of patients with pharmaco-resistant epilepsy a variety of diagnostic imaging tools are available, but despite extensive investigations, it is not always possible to accurately identify epileptic structures [3,4]. EEG-fMRI is a recent, valuable method that combines the good temporal resolution of EEG and the good spatial resolution of fMRI. It defines the irritative zone by studying the brain’s hemodynamic changes related to IEDs [5,6]. In focal epilepsy it has already been shown that the BOLD response localizes brain areas responsible for IEDs [7,8] and a good correlation between the BOLD response and the epileptic lesions could be seen [9,10]. Furthermore, the position of the positive BOLD response within the resected area was related to a good postoperative outcome [11]. EEG-fMRI resulted in additional clinical information in patients formerly rejected for epilepsy surgery [12,13]. Based on this new information some patients were surgically reevaluated and successfully operated [12,13].

Nonetheless fMRI is still not widely used in clinical practice. Up to 30% of EEG-fMRI studies are negative not showing any BOLD response [8]. Additionally BOLD responses are often hard to interpret and widespread or multifocal. While negative studies can be partly explained by a lack of epileptic spikes during the measurement or a bad signal-to-noise ratio, widespread BOLD responses are harder to interpret. Increased noise generated by technical artifacts, erroneous hemodynamic response functions (HRF) or improper statistical methods are partly made responsible [14]. An often discussed issue in this context is the thresholding of fMRI data as it influences the sensitivity of the method. Given that IEDs tend to rapidly propagate, widespread BOLD responses are in addition often regarded as propagated activity. Studies using fMRI combined with continuous EEG source imaging and those investigating early BOLD responses support this hypothesis [15–17].

In recent years fast fMRI sequences have been developed. The thereby achieved high temporal resolution increases signal to noise ratio and sensitivity of fMRI as well as the possibilities for investigating the time course of the BOLD response. In particular, a fast fMRI sequence called Magnetic Resonance Encephalography (MREG) [18,19] uses undersampled, single-shot k-space trajectories and a 32-channel receiver coil to acquire a 3D whole brain fMRI data with a temporal resolution of 100 ms and a spatial resolution of 4–5 mm. A former study could already demonstrate the higher sensitivity of MREG compared to the classical EPI method in patients with focal epilepsy [20]. IED related BOLD responses had higher t-values and better concordance with the EEG topography. Nevertheless some patients showed widespread BOLD responses, which were unclear in nature. The present study aims to improve understanding of the BOLD responses observed with MREG in patients with focal epilepsy. It first investigates whether widespread and multifocal positive or negative BOLD responses might be partly the result of large inter-spike variability when averaging IEDs for the analysis and then correlates the EEG IED extent with the associated positive and negative BOLD extent. Lastly, it explores the influence of EEG resolution on the interpretation of the positive and negative BOLD responses. All analyses aim to test the hypothesis that widespread BOLD in MREG do not reflect artifacts or unspecific metabolic changes but highlight structures within the epileptic spike generating network.

## Methods

Patients with focal epilepsy who stayed at the Epilepsy Centre Freiburg in the context of further diagnostic or treatment and met the following criteria were included in this study:

1. Minimum age of 6 years
2. > 3 IEDs in 20 minutes on EEG outside the scanner
3. No contraindications for MRI scanning

Clinical data were collected from the patient records. All patients gave written informed consent. On behalf of the minors written informed consent was given from the guardians. The study was approved by the Research Ethics Committee of the University Medical Center Freiburg.

### Data acquisition

EEG was continuously recorded inside the MRI scanner with an MR-compatible EEG-system (Brainproducts Co., Munich, Germany). 64 Ag/AgCl electrodes were placed on the patients head using an electrode cap (Easycap, Herrsching, Germany) with FCz as reference electrode. Electrode impedance was kept below 10 kΩ. Electrode cables and the patients head were immobilized with foam pads. Recording and storage of the EEG signal was carried out with the program `Brain Vision Recorder´ (Brainproducts Co., Munich, Germany). Data was transmitted via an optic fiber cable from an amplifier (5 kHz sampling rate, 0.016–250Hz band-pass filter) to a computer located outside the scanner room.

MREG recordings were continuously acquired during resting state using a 3 T (Tesla) scanner (Trio Tim, Siemens Healthcare, Erlangen, Germany). First an anatomical 3D, T1-weighted dataset (MPRAGE: TR = 2200ms, TE = 2.15ms, FOV = 256mm, 256×256 matrix, 160 sagittal slices, 1mm slice thickness) was performed for co-registration with functional images. MREG acquisitions were conducted for at least 20 minutes and up to 40 minutes, depending on patient compliance (TR = 100ms, TE = 20ms, FOV = 192mm, 64 × 64 × 64 matrix, flip angle = 15°, 12800 to 25600 volumes (time points), 32-channel head receiver coil). Electrocardiogram (ECG) and respiration were recorded simultaneously during the scan.

### EEG processing

Brain Vision Analyser software (Brainproducts, Munich, Germany) was used for offline artifact correction and EEG filtering. Gradient artifact and pulse artifact were removed by average artifact subtraction [21], followed by Independent Component Analysis [22,23]. IEDs were marked using a bipolar montage (double banana) by a board certified neurophysiologist (JJ) according to spatial distribution and morphology so that different types of discharges in one patient were analyzed separately. Timing with respect to the ECG was used to exclude IED-like residual pulse artifacts.

### Processing of MREG Data

First the MREG images were reconstructed from the raw data of the multiple receiver coils [19]. The fMRI images were motion corrected (coregistration with the first volume using MCFLIRT) and smoothed (Gaussian kernel, FWHM = 6mm) using FSL software (FMRIB`s Software library) [24]. Statistical analysis using the general linear model was performed using the software fMRIstat [25]. Cardio-respiratory regressors, motion parameters and the scanner drift were included as confounds in the model. To account for the high temporal resolution of MREG a 5^th^-order autoregressive (AR) was used to model the noise term in the general linear model (GLM) [20]. To account for possibly different HRF delays, four different HRFs were used with peak times at 3, 5, 7 and 9 seconds after the event [26]. For each voxel the highest absolute t-value among those four was taken to generate a single combined map. Responses were rated as significant if at least 7 contiguous voxels were present with |t|> 3.5 (p = 0.05, corrected for multiple comparisons at the cluster level [25] and an additional Bonferroni correction due to the 4 analyses).

### Further analysis

For further EEG analysis the ASA software (ANT Software BV, Enschede, Netherlands) was used. Spike peak correction was performed to automatically move the manual markings to the time of highest negativity. All marked IEDs within one IED set were averaged and EEG voltage maps were generated using a common average montage.

#### Validation of MREG by other established imaging methods

For validation of MREG an experienced neuro-radiologist (IM) visually compared the detected positive or negative BOLD responses to structural lesions in magnetic resonance (MRI) and statistically significant metabolic areas in positron emission tomography (PET) and single photon emission computed tomography (SPECT).

#### Correlation between IED and BOLD extent

**Fig 1:**
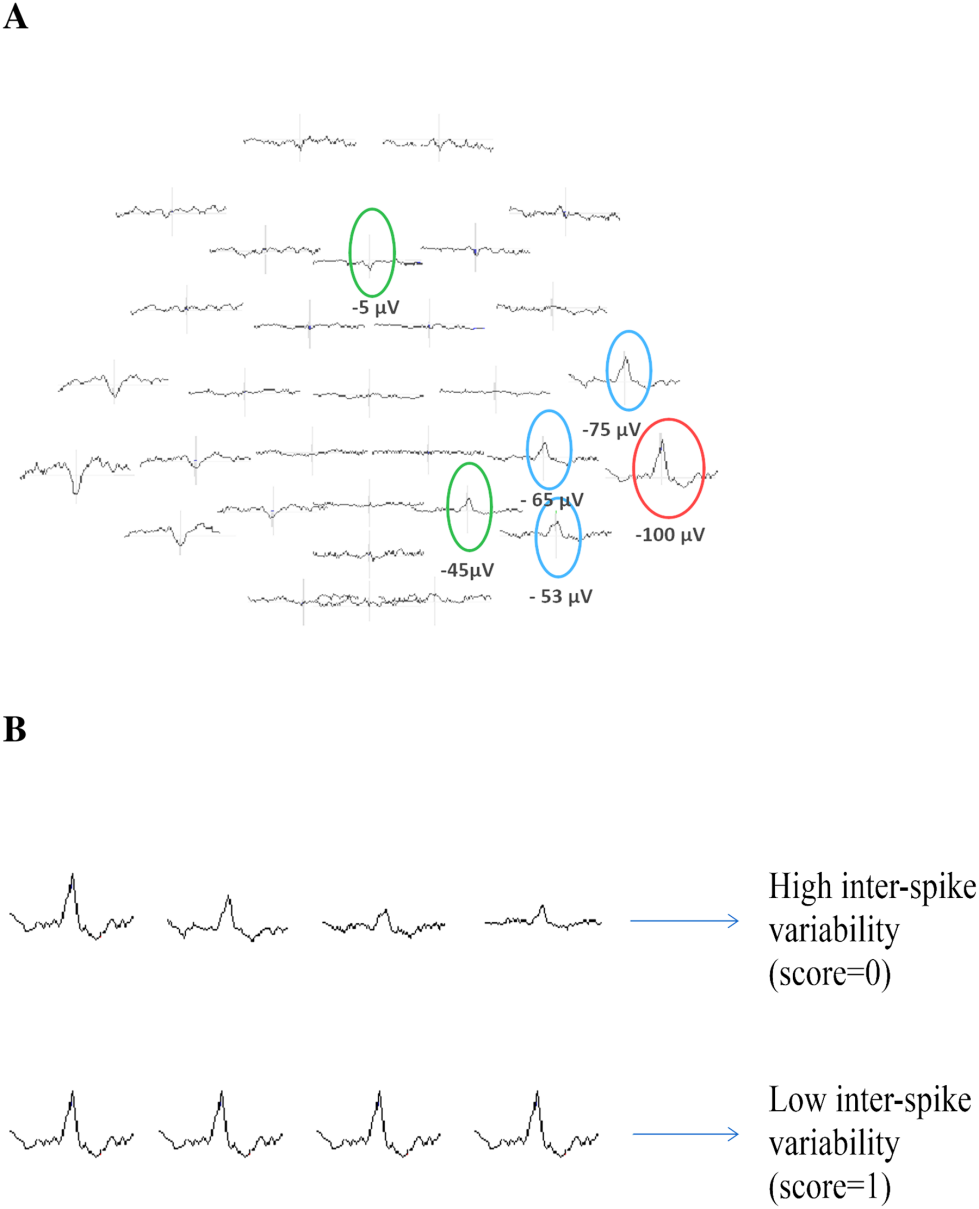
Exemplary illustration of the used methods. (**A**) Determination of IED extent. The red circle indicates the IED with the highest negativity, the blue circle indicates IEDs with at least 50% of this negativity and the green circle indicates IEDs that did not meet the inclusion criteria. (**B**) Representation of possible inter-spike variability within one IED set.

IED extent was measured in the 64-channel EEG voltage map by identifying the electrode with the highest negativity (in μV) and all other electrodes which showed at least 50% of this negativity (Fig 1). The included electrodes were counted and the resulting number taken as IED extent. The total extent of BOLD volume measured as the entire region of activated voxels in cm³ as well as the total number of BOLD clusters was determined using MREG data and then both separately correlated with the IED extent using a Spearman Correlation (Table 1). This was performed for positive and negative BOLD responses separately.

**Table 1:** IED and BOLD extent.

Patient	Spiketype	Electrode with maximum spike negativity	Electrodes with ≥ 50% of maximum spike negativity	Number of involved electrodes	Positive BOLD volume in cm³	Number of positive BOLD clusters	Cluster with max T-value
**1**	Spike 1	TP10	TP8, FT8, FT6, T8, CP6	5	3928	18	308
	Spike 2	F8	FP2, AF8, F6, FT8, T8	5	3688	17	2079
**2**	Spike 1	FP2	FP1, AF4, AF8, FZ, F2, F4, F6, CPz	8	4684	24	3781
**3**	Spike 1	C4	FC6	1	1783	17	446
	Spike 2	F3	FP1, AF7, AF3, F7, F5, F1, F2, F4, FT9, TP7, P3, O1	12	4065	8	2961
**4**	Spike 1	FP2	FP1, F3, F4,	3	1643	8	427
	Spike 2	C1	FP1, AF7, AF3, F7, F5, F3, FT7, FC5, FC3, FC1, C5, C3, Cz, CP2, CPz, CP1, CP3, Pz	3	3740	31	327
	Spike 3	FP2	FP1, F4	2	2152	15	898
**5**	n.a.						
**6**	Spike 1	C3	C5, C1, CP3, FC1, FC3	5	744	16	59
	Spike 2	TP9	FT9, FT7, FC5, CP5	4	8879	28	8107
	Spike 3	FT7	FT9, FC5, T7, C5, TP9, TP7, CP5, POz, TP10	9	2285	17	1152
	Spike 4	C5	T7, C3, FT9, FT7, FC5, FC3, F7, AF7, TP7, CP5, CP3	11	7156	21	6687
**7**	Spike 1	T7	FT9, FT7, TP9	3	2382	18	823
**8**	Spike 1	FP2	FP1, F3, C3	3	741	18	114
	Spike 2	POz	TP10, CP6, CP2, CP1, CP5, TP9, FC5, FC6	8	1453	14	319
**9**	Spike 1	F3	C3	1	2320	22	1084
	Spike 2	POz	TP9, TP10, CP5, CP1, CP2,CP6, FC6, FC5	8	3313	20	2017
	Spike 3	TP9	TP10, CP6, CP5, CP2, FC5, FC6, Poz	7	358	10	154
**10**	Spike 1	F7	AF7, AF3, F5, F3, FC3, FC5, FT7, FT9	8	2020	25	12
	Spike 2	TP9	FT9, FT7, T7, TP7, P7	5	3118	40	25
	Spike 3	TP10	T8, TP8,	2	2110	28	795
**11**	Spike 1	T8	TP10, CP6, P8, C6, FT10, FT8, FC6, F8, Oz, O2	10	1920	36	725
**12**	Spike 1	FT9	AF4, AF7, FPz, FP2, F7, F5, F3, FT7	8	3970	37	270
	Spike 2	FP1	F3, F4, C3, C4	4	278	15	52
**13**	n.a.						
**14**	Spike 1	AF7	FP1, FPz, FP2, AF3, AF4, F7, F5, F3, F1, FZ, F2, F4, FT9, FT7, FC5, FC3, FC1, FC2,	18	11264	71	2859
**15**	Spike 1	FT10	FT8, FC6, FC4, F8, F6, F4, AF4, FP2, T8, C6	10	8401	28	5567
	Spike 2	TP9	FT9, TP7,T7, P7, P5, P3	6	12966	19	12537
	Spike 3	P4	P2, P6, CP2, PO4	4	0	0	0
**16**	Spike 1	FPz	FP1, FP2, AF7, AF3	4	1602	19	187
	Spike 2	F2	FP1, FP2, AF7, AF3, AF4, AF8, F7, F5, F3, F1, Fz, F4, F6, F8, FT7, FC5, FC3, FC1, FC2, FC4, FC6, FT8, C5	23	1681	12	558
**17**	Spike 1	F5	FP1, FP2, AF7, AF3, AF4, F3, F1, F2, FC5, FC3, FC1, C3, Cz, CP3	14	2883	16	1554
	Spike 2	FT9	FP1, FPz, FP2, AF7, AF3, F7, F3, FT7	8	3825	24	1378

AF, anteriofrontal; C, central; CP, centroparietal; F, frontal; FC, frontocentral; FP, frontoparietal; FT, frontotemporal; n.a., not available; O, occipital; P, parietal; PO, parietooccipital; T, temporal; TP, temporoparietal; z, central

#### Influence of inter-spike variability on BOLD response

Rather than averaged IEDs, every single spike of one IED set was considered here. The IED variability across spikes was quantified by correlating the individual spatial topographies (using the 64-channel EEG) of each IED with the previously generated average spatial topography across all IEDs of a given set. Coefficient of correlation was computed with MATLAB. The mean correlation across all IEDs was then used as a measure of variability. The higher the mean correlation the more similar are the single spikes of one IED set. A value of 1 would therefore indicate that all individual maps are identical and correlate perfectly with the average map, while a lower score indicates a higher variability across maps (Fig 1). It was then investigated whether there was a difference in this variability score between IED sets with concordant positive or negative BOLD responses, and IED sets without concordant positive or negative BOLD responses. Both groups were compared with a t-test (level of significance α<0.05).

#### Comparison 32/64-channel EEG

**Fig 2:**
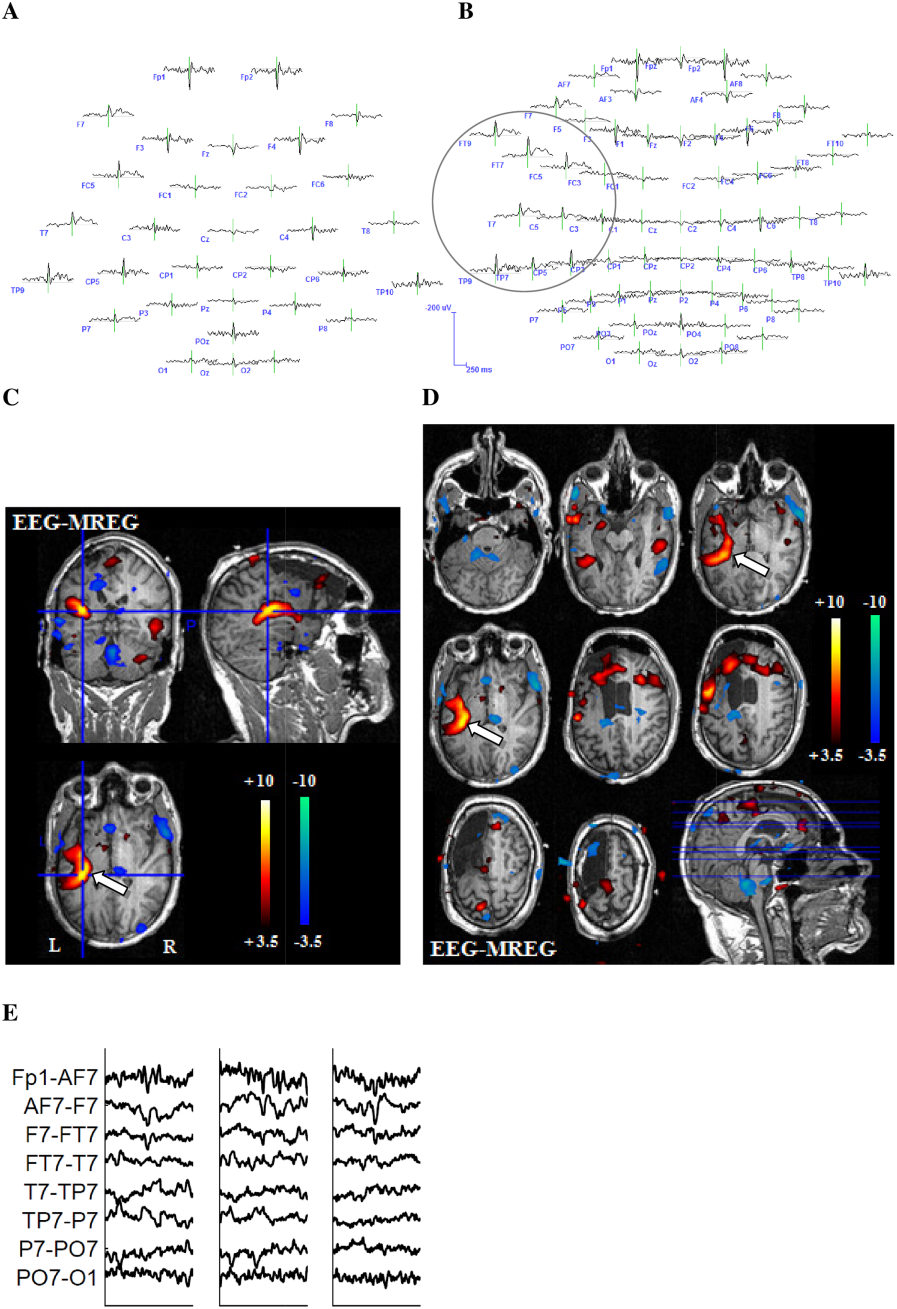
Example 1 for better representation of the BOLD-topography with 64 electrodes. 27-year-old patient (patient 6, study 3) with left structural frontal lobe epilepsy. The patient underwent a previous operation with frontal resection, which showed a FCD type 2a. No seizure freedom could be achieved through operation. This is a typical example for the superiority of the 64-channel-EEG. The 32-channel EEG reveals a left temporoparietal IED area (**A**). The 64-channel EEG shows that left frontotemporal regions are as well included in the IED area (**B.**) The extensive positive BOLD response on the left hand side exactly reflects the IED distribution from temporoparietal to frontotemporal as shown in the 64-channel EEG. The white arrow indicates the relevant positive BOLD response (**C** and **D**). (**E**) shows different IEDs of one IED set.

The before generated EEG voltage maps were examined for correspondence of irritative zone and positive or negative BOLD responses (positive and negative). This analysis was performed using the original 64-channel EEG voltage maps as well as a conventional 32-channel electrode subset (Fig 2). Included electrodes in the 32-channel EEG were Fp1, Fp2, Fz, F3, F4, F7, F8, FC1, FC2, FC5, FC6, Cz, C3, C4, T7, T8, CP1, CP2, CP5, CP6, TP 9, TP 10, Pz, P3, P4, P7, P8, POz, Oz, O1, O2. Additionally included in the 64-channel EEG were the electrodes FPz, AF3, AF4, AF7, AF8, F1, F2, F5, F6, FC3, FC4, FT7, FT8, FT9, FT10, C1, C2, C5, C6, CPz, CP3, CP4, TP7, TP8, P1, P2, P5, P6, PO3, PO4, PO7, PO8. For each IED set, both voltage maps were visually compared to evaluate which one better represents the irritative zone as well as which one better reflects the area of the positive or negative BOLD response using the following criteria:

- Between both montages the noise level was compared. Data was considered as noisy if more than 10% of EEG channels showed artifacts or unidentifiable wave forms at the time of the IED, or if EEG and dipole maps were unable to identify one clear dipole
- The electrode with the highest negativity was classified as IED origin. Positive or negative BOLD responses were rated as concordant when this highest negativity and the highest BOLD were located in the same brain lobe
- The extent and location of EEG IED spread, namely those channels in which a negative peak was visible at the time of the IEDs, were visually compared with the positive or negative BOLD extent and location

## Results

17 patients could be included in the study. Patient age was between 9 and 71 years (mean age: 27.3years). All clinical information on patients is summarized in Table 2. 15 patients showed IEDs and 32 IED sets were identified. Patient 5 and patient 13 did not show any IEDs during MREG. In all 32 IED sets, positive as well as negative BOLD responses could be identified.

**Table 2:** Clinical information.

Patient	Age	m/f	Age of onset	Epilepsy classification	Seizure types	Inter-ictal EEG	MRI	PET/SPECT	AED	Surgery outcome
1	36	m	7 y	Structural TLE	CPS	F+T right	MTS right	n.a.	LTG LCM	n.a
2	26	m	7 y	Structural TLE	CPS	FT bilateral > FT left	Hypothalamic hamartoma	PET: discrete hypometabolism P, T right	LEV OXC LCM	Seedtherapy:not seizure free
3	17	f	16 y	Structural TLE	CPS	T + TP right; T left	Unclear mass in the left superior T gyrus	n.a.	OXC	n.a.
4	17	m	13 y	Unclear	SPS/CPS GTCS	PO bilateral	Normal	PET: discrete hypometabolism P left	LTG OXC	n.a.
5	27	f	16 y	FLE of unclear origin	CPS/GTCS	F bilateral	Normal	PET: normal	LTG LEV	n.a.
6	27	m	11 y	Structural FLE	SPS/CPS GTCS	FC, FT left	Surgical cavity left F	PET: extensive hypometabolism TP left	LEV OXC	surgery: not seizure free
7	12	m	9 y	Structural FLE	CPS	F bilateral; T left	Cavernoma F right	n.a.	none	n.a. (surgery) out?
8	9	m	4 y	Structural FLE	SPS/CPS GTCS	FC bilateral T bilateral	Extensive right polymicrogyria	PET: hypometabolism F, P left	LEV VPA	n.a.
9	28	f	11 y	FLE of unclear origin	SPS/CPS GTCS	FP-FC right TP right	Unclear lesion right, F including insular cortex	PET/SPECT: hypometabolism F right	LTG LCM	surgery: not seizure free
10	71	m	70 y	Structural TLE	CPS	T pole right	Cystic tumor mesio temporal left	n.a.	VPA	n.a. surgery?
11	31	f	31 y	TLE of unclear origin	CPS	T right	n.a.	n.a.	OXC	n.a.
12	60	m	40 y	unclear	CPS	T bilateral F bilateral	Defect/gliosis T pole left	PET: hypo metabolism T left	OXC	n.a.
13	13	m	3 y	Structural TLE	CPS/GTCS	CP bilateral T bilateral	MTS left and gliosis TP	n.a.	VPA LEV	n.a.
14	23	m	n.a.	unclear	SPS/CPS GTCS	T right > T left F left O right	abnormal gyration O right	n.a.	LTG CLO	n.a.
15	39	f	23 y	TLE of unclear origin	CPS	T bilateral	Hippocampus malrotation left	PET: normal	LTG	n.a.
16	13	f	7 y	unclear	CPS/GTCS	FC bilateral; F left	FCD medial-basal right	PET: normal	CAB VPA	n.a.
17	15	m	12 y	unclear	SPS/CPS GTCS	FC bilateral P left	n.a.	PET: hypometabolism F left	LTG	n.a.

AED, anti epileptic drug; C, central; CAB, carbamazepine; CLO, clobazam; CPS, complex partial seizure; f, female; F, frontal; FC, frontocentral; FLE, frontal lobe epilepsy; FP, frontopolar; FT, frontotemporal;GTCS, generalized tonic clonic seizure; LCM, lacosamide; LEV, levetiracetam; LTG, lamotrigine; m, male; MTS, mesial temporal sclerosis; n.a., not available; O, occipital; OXC, oxcarbazepine; P, parietal; SPS, simple partial seizure; T, temporal; TLE,temporal lobe epilepsy; TP, temporoparietal; VPA, valproate acid; y, year

### Validation of MREG by other established imaging methods

**Fig 3:**
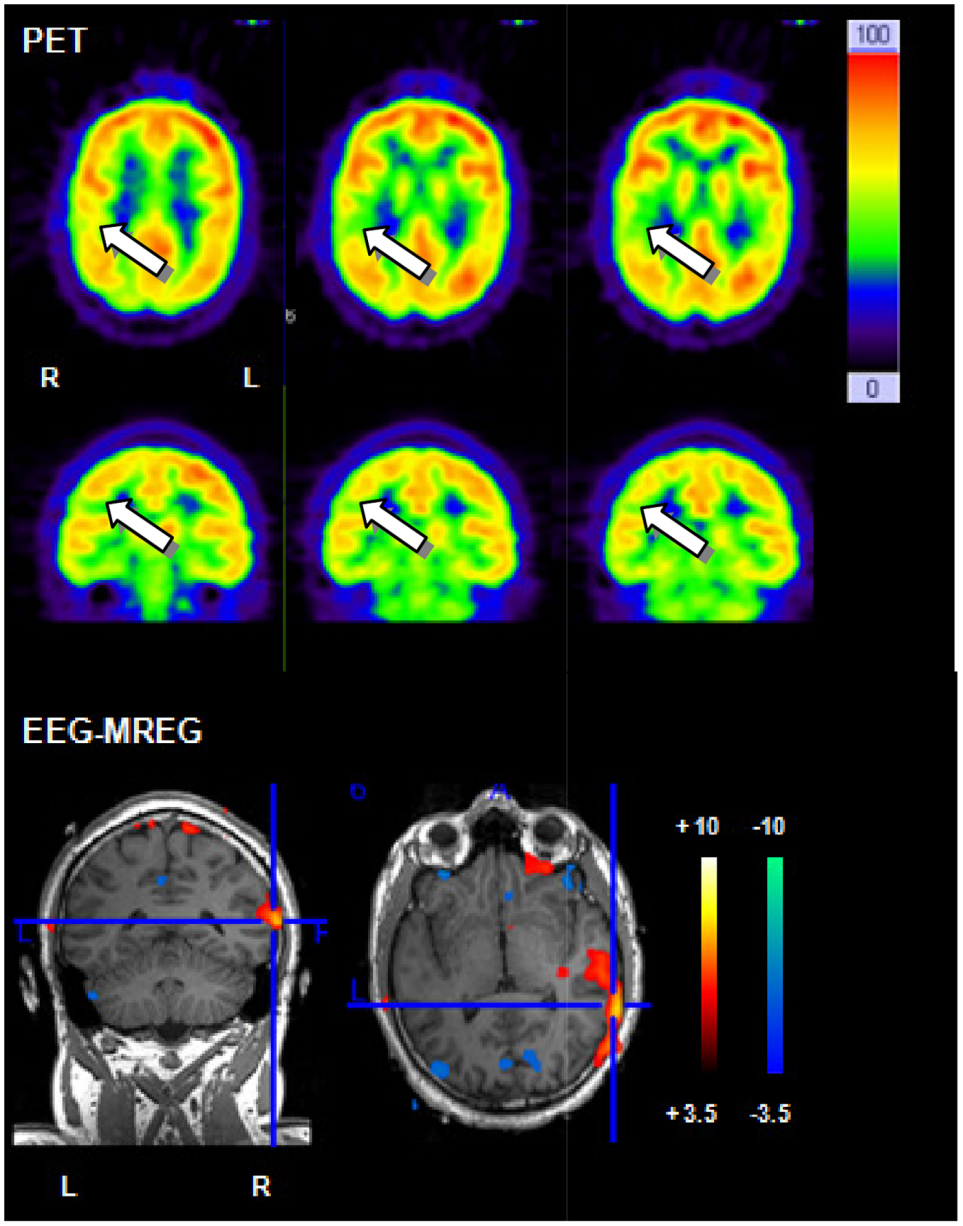
Example for correlation between PET and positive BOLD response. 26 year old patient (patient 2) with structural epilepsy. PET shows a discrete temporal and parietal hypometabolism. The strongest positive BOLD response in MREG is located temporal on the right hand side and therefore shows a good correlation with PET.

In 12 (24 IED sets) patients a clear lesion in the MRI could be found. FDG-PET studies were conducted in 9 patients but visual assessment of FDG PET identified regions of focal hypometabolism in only 7 patients. Ictal SPECT was conducted in one patient and a region of focal hyperperfusion could be identified. Topographic correlation between positive BOLD response and MRI lesion was found in 10 /12 patients. Positive BOLD responses were concordant with PET results in 5/7 patients and with ictal SPECT results in 1 patient. One example with concordant positive BOLD responses and PET results is displayed in Fig 3.

### Correlation between IED extent on scalp EEG and BOLD extent

**Fig 4:**
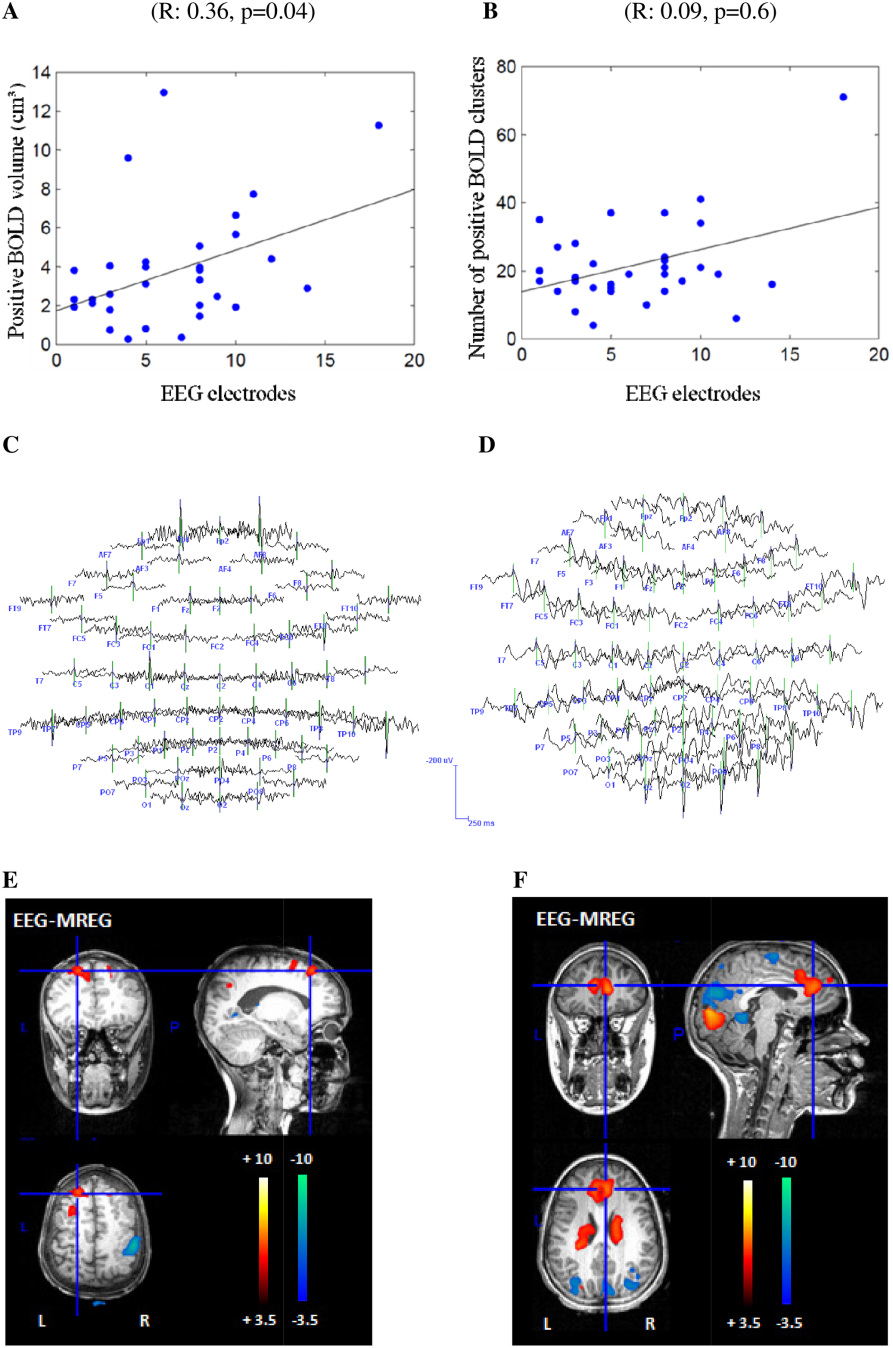
Representation of the positive correlation between IED and BOLD extent. The x-axis shows the in the IED extent included number of electrodes. The y-axis once shows the volume of positive BOLD responses (**A**) and once the number of positive BOLD clusters (**B**) in MREG. Patient 8, study 1 reveals a restricted frontal IED area in the EEG (**C**), concordant to the left focal positive BOLD response in MREG (**E**). Only few other clusters are visible, all of them focal. In comparison, patient 16, study 2 reveals a widespread IED area in the EEG (**D**) with the highest negativity over F2 and multiple, widespread BOLD clusters in MREG (**F**). However, the BOLD response located at the front is concordant to the most negative IED area over F2 and the MRI results.

The more widespread the IED area the more voxels were covered by positive BOLD (R: 0.36, p = 0.04) (Fig 4). No correlation was found between the IED extent the number of positive BOLD clusters (R: 0.09, p = 0.6) and the volume (R: 0.28, p = 0.1) or the number of negative BOLD clusters (R: 0.05, p = 0.8). Results are listed in detail in Table 1. We also computed the correlation between the IED extent and the volume of the cluster with peak T-value [27,28]. No significant correlation was found with the cluster volume with peak negative T-value (R: 0.29, p = 0.1), although a trend could be detected with the cluster volume with peak positive T-value (R: 0.34, p = 0.06).

### Influence of IED variability on BOLD concordance

Overall the coefficients of correlation of variability varied between 0.12 and 1. For the 25 IED sets with concordant positive or negative BOLD responses, variability between the single spikes within one IED set showed a mean value of 0.71 (SD: +/-0.27). Variability of the 7 IED sets without a concordant positive or negative BOLD response showed a mean value of 0.48 (SD: +/-0.16). Inter-spike variability was significantly higher for IED sets without than with concordant BOLD responses (p = 0.04). An example for different spikes of one IED set is shown in Fig 2.

### Comparison 32/64-channel EEG

**Fig 5:**
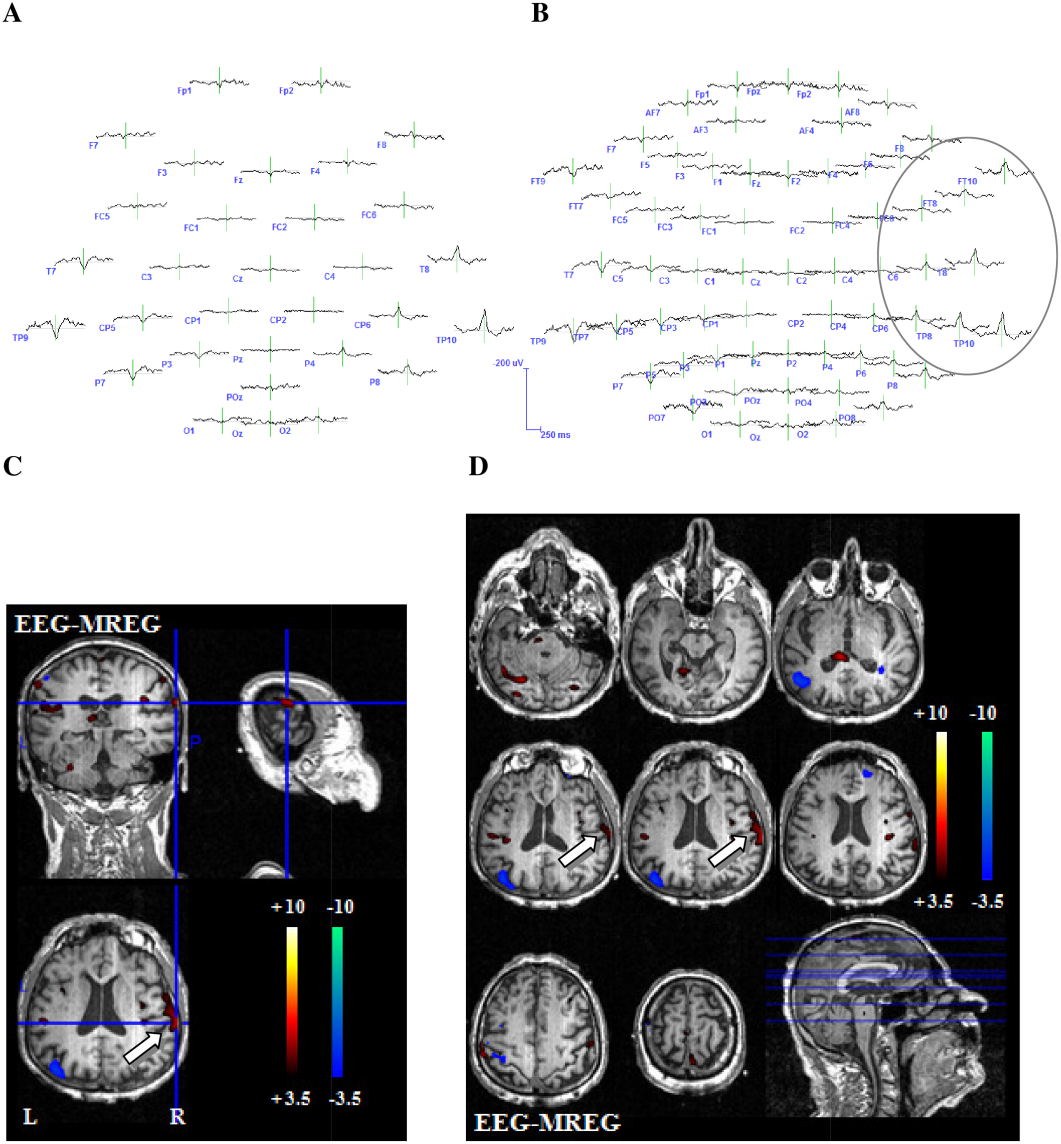
Example 2 for better representation of the BOLD-topography with 64 electrodes. 71-year-old patient (patient 10, study 3) with structural temporal lobe epilepsy. Another example for the predominance of the 64-channel-EEG. The 32-channel EEG reveals a temporoparietal IED area (**A**). The 64-channel EEG shows that the IED area as well spreads into frontotemporal regions (**B**). The positive BOLD response on the right hand side represents the IED distribution from temporoparietal to frontal regions as shown in the 64-channel EEG. The white arrow indicates the relevant positive BOLD response (**C** and **D**).

For the 64-channel EEG, correlation between positive BOLD response and IED topography could be found in 19 IED sets and a correlation with a negative BOLD response in 7 IED sets. For the 32-channel EEG, correlation between positive BOLD response and IED topography could be found in 18 IED sets and a correlation with a negative BOLD response in 7 IED sets. In 16 IED sets visual localization was better in the 64- than the 32-channel EEG. In all remaining IED sets no difference between the two EEG montages could be found. Reason for the superiority of the 64-channel EEG was the location or spread of IEDs in temporal brain areas which are not well covered by the 32-channel EEG, or whenever IEDs had their highest negativity over electrodes not represented in the 32-channel EEG. In a few cases a larger number of electrodes lead to more noise in terms of unclear, widespread and multifocal dipoles within the dipole maps and an impeded identification of the IED area. Out of the 16 IED sets profiting from high resolution EEG, in four improved concordance with the strongest positive and in 2 with the negative BOLD change (examples Figs 2 and 5) were observed. Only in one IED set the 32-channel EEG better reflected the negative BOLD response. Reason for the superiority of the 64-channel EEG was at all times the location or spread of the BOLD response in temporal brain areas. All results are depicted in detail in Table 3. BOLD-topographies of all patients can be found in the supporting information (S1–S4 Figs).

**Table 3:** Detailed representation of BOLD effects and comparison between 32/64 channel EEG.

		positive BOLD			negative BOLD		
	Spike topography	32 electrodes	Comparison 32/64	64 electrodes	32 electrodes	Comparison 32/64	64 electrodes
**1**	TP right	+	=	+	−		−
	T right	+	=	+	−		−
**2**	Fp right	+	=	+	+	=	+
**3**	CP right	−		−	−		−
	F left	+	=	+	−		−
**4**	Fp right	+	=	+	−		−
	C left	−		−	+	=	+
	Fp right	+	=	+	−		−
**5**	n.a.	n.a.	n.a.	n.a.	n.a.	n.a.	n.a.
**6**	C left	+	=	+	−		−
	TP left	+	=	+	−		−
	FT left	+	<	+	−		−
	C left	+	=	+	−		−
**7**	T left	−		−	−		−
**8**	Fp right	+	=	+	−		−
	PO central	+	=	+	−		−
**9**	F left	−		−	+	=	+
	PO central	−		−	+	=	+
	TP left	+	=	+	−		−
**10**	F left	−		−	−		−
	TP left	+	=	+	−		−
	TP right	−	<	+	−		−
**11**	FT right	+	<	+	−		−
**12**	FT left	−		−	+	>	−
	Fp left	−		−	+	=	+
**13**	n.a.	n.a.	n.a.	n.a.	n.a.	n.a.	n.a.
**14**	F left	−		−	−	<	+
**15**	FT right	−		−	+	<	+
	TP left	+	=	+	−		−
	P rigth	−		−	−		−
**16**	Fp central	+	<	+	−		−
	F right	+	=	+	−		−
**17**	F left	−		−	−		−
	FT left	−		−	−		−
	**Summary**	**18**	**4 (0/4)**	**19**	**7**	**3 (1/2)**	**7**

C, central; CP, centroparietal; F, frontal; FC, frontocentral; FP, frontoparietal; FT, frontotemporal; n.a., not available; P, parietal; PO, parietooccipital; T, temporal; TP, temporoparietal; +, concordance between BOLD and spike topography; -, no concordance between BOLD and spike topography; <, predominance of the 64-channel-EEG; >, predominance of the 32-channel EEG; = or free field, there was no difference between the 32- and the 64-channel map.

## Discussion

In all patients and IED sets positive as well as negative BOLD responses were found with MREG. This study therefore could confirm the high sensitivity of MREG for the occurrence of BOLD responses, as has already been shown previously [20]. However, as MREG is a newly developed method, the observed BOLD needs further verification, which we could provide in this study by demonstrating a correlation between extent of scalp IEDs and positive BOLD responses. Moreover higher density EEG suggested a clearer correlation between EEG IED voltage maps and areas showing increased BOLD. Last but not least evidence suggests that large spike variability of IEDs generated over the same brain area might be an important reason for discordant BOLD effects.

### Correlation between BOLD response and other diagnostic measures

In 83% of the patients (10/12 patients) a correlation between the lesion found in MRI and the positive BOLD response existed. Other studies showed similar results, with 61.5–85% [9,10]. We could achieve such a high correlation even though our patient collective was very heterogeneous in comparison to the other studies. We included all patients with focal epilepsy regardless of their underlying pathology, among them many patients with epilepsy of unknown etiology or lesions with unclear epileptogenicity. For the latter we would not necessarily expect a correlation between positive BOLD and lesion. Correlation between PET and positive BOLD was found in 71% of the patients (5/7 patients). This is consistent with other studies with sensitivities of 73%-100% [12,29,30]. The two patients in our study without concordance of any IED type and PET were as well the only ones with normal MRI. Overall, in 10 patients the positive BOLD response could be confirmed by at least one other diagnostic in addition to EEG. The comparison between EEG-MREG and other methods therefore supports the hypothesis that positive BOLD responses in fact represent the irritative zone and epileptogenic areas just as the other methods do.

### Correlation between IED and BOLD extent

Our study statistically revealed a direct connection between the extent of the IED as calculated from the scalp EEG and positive BOLD extent. Focal IEDs correlated with focal positive BOLD responses while widespread IEDs correlated with widespread positive BOLD responses. Two other recently published studies came to the same conclusion, one by only visually estimating this connection without calculating it [31] and the other one by comparing the measured BOLD volume related to focal spikes with widespread bilateral synchronous discharges in frontal lobe epilepsy [32]. This finding could suggest that widespread positive BOLD responses are not simply artifact or unspecific reactions, but probably correctly reflect the broad network of propagating IEDs. Studies with continuous EEG source localization have been able to support this assumption [15,16]. By gaining information regarding the time course of the IEDs, different BOLD responses could be related to different parts of the IED network and maybe even areas of seizure onset or propagation.

This information is increasingly relevant for fast fMRI sequences like MREG, for two reasons. First, increased sensitivity of the sequence might lead to more extensive BOLD responses that require interpretation, second, the high temporal resolution might provide the possibility to analyze time courses of widespread BOLD changes. The first consideration is supported by Gonzalez and colleagues [33] who indicated that BOLD responses to a presumably focal task might actually be present in the entire brain, and may be detected given a sufficiently sensitive acquisition whereby it becomes increasingly unclear which brain areas are important. It also poses the question whether thresholds defined and validated for classical fMRI apply for MREG as well or if they reveal too many unspecific BOLD responses. It nevertheless appears that the often widespread positive BOLD regions observed with MREG seem to represent epileptic networks. Whether they are clinically relevant for diagnostic purposes will have to be further evaluated in studies involving the postsurgical outcome or results of intracranial EEGs, as already performed for classical fMRI [12,34]. MREG as well provides the possibility to investigate the temporal development of the BOLD response. With its high temporal resolution, it becomes possible to track the precise onset of the BOLD response for each individual IED. A detailed analysis and interpretation of this time course of the BOLD response could allow a differentiation between IED generation and propagation. It will however be necessary to develop new analysis methods to really profit from the newly gained temporal resolution. Until then analyzing the BOLD with the highest t-value can be used to find a focal source, as was done for clinical correlations in the present study.

### Spike variability

Another open question in EEG-fMRI interpretation is the lack of concordant BOLD responses in cases with sufficient IEDs during the measurement [35]. Our results suggest that inter-spike variability might be the reason for these negative studies. It is of course important to correctly mark IEDs, preselect congruent IEDs and define different IED sets, to ensure a successful EEG-fMRI analysis. However, to clearly identify IEDs in the EEG poses a challenge even to experienced epileptologists, and even very similar looking IEDs may have different degrees of variability, as revealed in this study. Therefore the visual identification of IEDs in this study is error-prone and has its limitations. An approach, to guarantee that all similar IEDs of one IED set are detected and all unclear IEDs are excluded, could be the application of `consensus´ IEDs or templates [13,36], with the former defined as being marked with high agreement by two independent experts. Also other forms of detecting IEDs are possible. One alternative method uses ICA to separate IEDs from the EEG background [37]. Another recently published study applied a spike sorting algorithm to classify the before visually identified events and hereby achieved a higher rate of correspondence between IED classes and BOLD response [38]. Another issue lies in the definition of IED extent on the EEG. Scalp EEG amplitudes are reference dependent; we used an average reference and a 50% amplitude threshold relative to the maximum spike peak to define the IED extent, but absolute voltage quantifications would also be possible when projecting the scalp topography to an infinity reference [39]. MREG also promises a different solution to the problem of inter-spike variability, as the high sensitivity of the method may allow the analysis of single IEDs generated over the same brain regions [20,40]. The future might therefore be to look at maps of a number of single IEDs and get an even more detailed picture of the different brain regions involved in the irritative zone with an emphasis on reproducible activation patterns.

### Comparison 32/64-channel EEG

In 50% of the IED sets the 64-channel EEG outperformed the 32-channel EEG. This is in agreement with previous studies [41,42] that have proven the advantage of high density EEG in spike source localization. In 37% of the cases in which the 64-channel EEG improved interpretation of the IED localization it also outperformed the 32-channel EEG in reflecting the area of positive or negative BOLD response. One main reason for the superiority of the 64-channel EEG were IEDs originating in temporal regions, which are not well covered by the 32-channel EEG. Other studies could show that in patients with temporal lobe epilepsy a large number of spikes will be missed whenever EEGs with a low number of electrodes are used [43]. Furthermore it could lead to a shift of the spikefield to more dorsal regions if the inferior chain of electrodes is missing [44]. Therefore a high density EEG should be chosen especially for patients with suspected temporal lobe epilepsy, or at least the additional temporal electrodes should be added to the classical 10–20 electrode arrangement. Another reason for the superiority of the 64-channel EEG in better depicting the irritative zone was whenever IEDs had their highest negativity over electrodes not represented in the 32-channel EEG. However this never led to a better reflection of the positive or negative BOLD response as the IED area normally involved more than one electrode and therefore only changed marginally. This result also reflects the fact that most of the time positive or negative BOLD responses in our study were not very focal. When comparing EEG and BOLD response a high spatial resolution in areas already covered by electrodes therefore seems less profitable. Nevertheless in EEG-fMRI a high spatial resolution becomes important when using detailed source localization methods whose results can then be compared with the location of the strongest BOLD [34,45]. However, a larger number of electrodes can also lead to more noise [41]. This could be observed in our study in a few cases as well. The irritative zone then seemed to be more widespread and its localization was impeded. But overall a clear superiority of the 64-channel EEG was evident.

A correlation between BOLD responses and EEG revealed a sensitivity of 81.2% (26/32 IED sets) with 19 IED sets showing a positive and 7 a negative BOLD response in the area of IED onset. The sensitivity is similar to former EEG-fMRI and MREG studies [7,8,13,20]. Even if some correlation between spike localization and negative BOLD was found, the predominant correlation was found with positive BOLD responses. A large majority of negative BOLD responses were found in distant areas, some of them showing large overlap with the default mode regions [46]. This has been previously described for EEG-fMRI in epilepsy and is increasingly found when using MREG [20]. Therefore it is likely that not all negative BOLD is directly representing the irritative zone, some of it however might be relevant as some studies on the time course of the hemodynamic response demonstrated that some negative responses after the spikes are preceded by positive responses prior to the spike in the same brain region [17]. Additionally, BOLD changes in white matter and CSF are generally attributed to physiological noise, which greatly affects fMRI data. Fast acquisitions such as MREG may however allow the development of new methods for physiological noise correction, which may alleviate this problem in the future.

### Methodological limitations

In this study we didn’t directly compare conventional fMRI with MREG, as already investigated in a previous study [20]. Conclusions related to the performance of higher density EEG and the relationship of IED extents seen in EEG and fMRI are therefore made in relation to fast fMRI, but could very well apply to conventional fMRI as well.

It still remains open which HRF to use for EEG-fMRI analysis. There is evidence that HRFs not always follow the same time series and that differences exist between patients, age or brain areas [47,48]. HRFs following focal epileptic spikes may differ from the standard HRF, a canonical HRF which follows short auditory stimuli [49]. This variability of HRFs in epilepsy patients could also be shown by other studies [50,51]. For statistical analysis we therefore used multiple HRFs as it has been shown that this can increase the sensitivity of EEG-fMRI in epilepsy [26]. Thereby even BOLD responses can be detected that strongly differ from the standard canonical HRF [49]. A recently published study using MREG defined a subject-specific HRF model which improved model accuracy and led to increased t-values and a larger size of activation foci [52].

MREG with its high temporal resolution and increased sensitivity may also be more vulnerable to false positive activations originating from noise. The 1st-order autoregressive model normally used in EPI resulted in false activations including all brain areas [20]. A 5th-order autoregressive model was therefore selected. The determination of the most suitable model however is still a part of research [53].

## Conclusion

EEG-MREG proved to be a valuable and sensitive tool for diagnostics in epilepsy and might be able to identify network structures of IEDs. While EEG alone can only show widespread IED activity, EEG-MREG opens the opportunity to have better localization of epileptic activity even in deep brain structures. Brain regions with the strongest positive BOLD effect show a good correlation with the IED focus, lesion and results from other neuroimaging methods. High inter-spike variability seems to impede good correlations between positive or negative BOLD and IED topography. To avoid this phenomenon in the future averaged IED analysis might be replaced by single IED analysis possible due to the increased sensitivity of MREG. In the next step MREG results should be evaluated in the context of intracranial EEG recordings and postsurgical outcome for further validation.

## Supporting Information

S1 Fig BOLD-topographies of patient number 1 to patient number 6 Spike 1. (TIF)

S2 Fig BOLD-topographies of patient number 6 spike 2 to patient number 10. (TIF)

S3 Fig BOLD-topographies of patient number 11 to patient number 17 spike 1. (TIF)

S4 Fig BOLD-topography of patient number 17 spike 2. (TIF)
