# Supplementary figures and images for: Concordance of Epileptic Networks Associated with Epileptic Spikes Measured by High-Density EEG and Fast fMRI
Source: PLoS One. 2015 Oct 23;10(10):e0140537. doi: 10.1371/journal.pone.0140537 (PMC4619722; doi:10.1371/journal.pone.0140537)

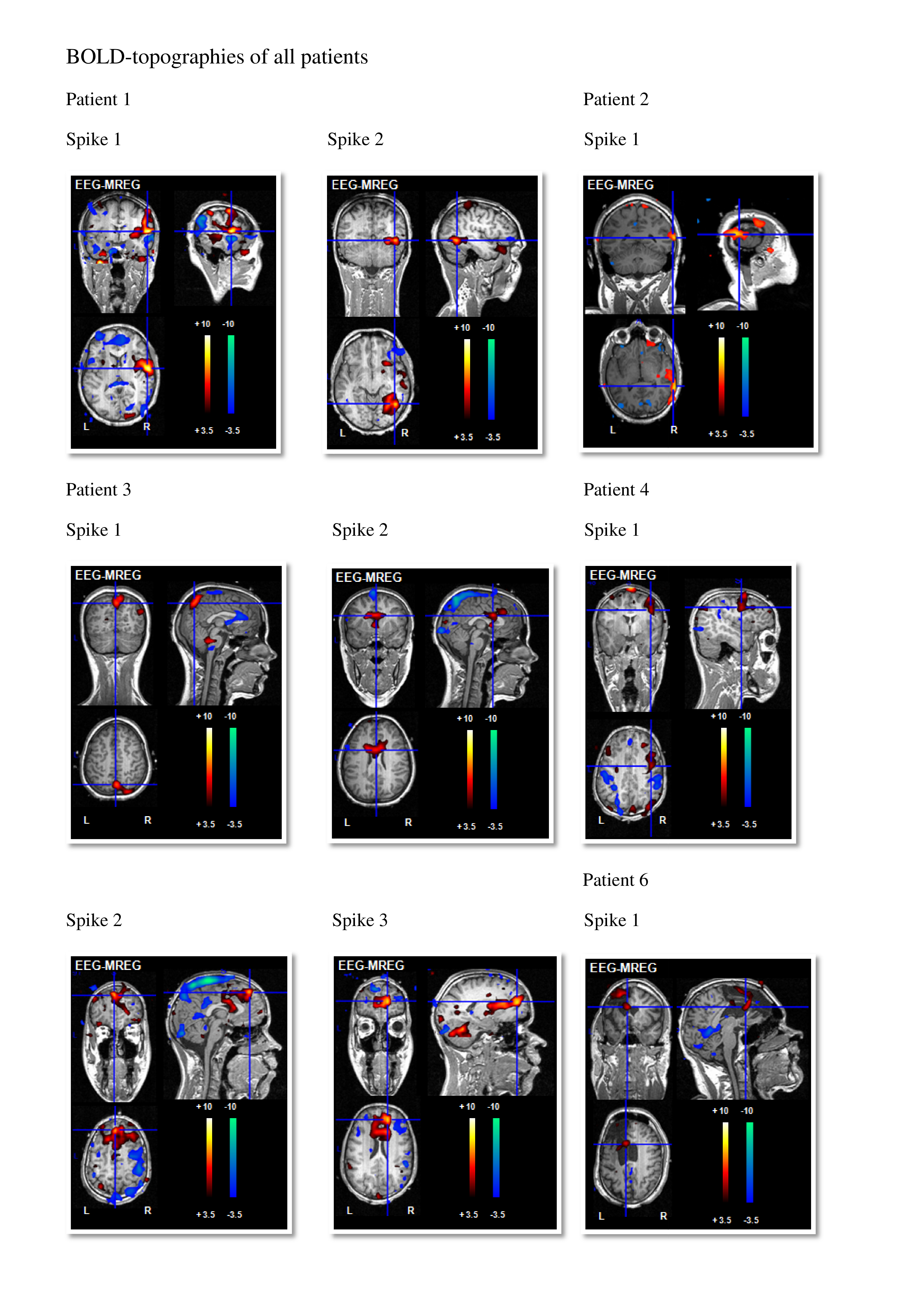

Supplement: S1 Fig — (TIF) [file pone.0140537.s001.tif]

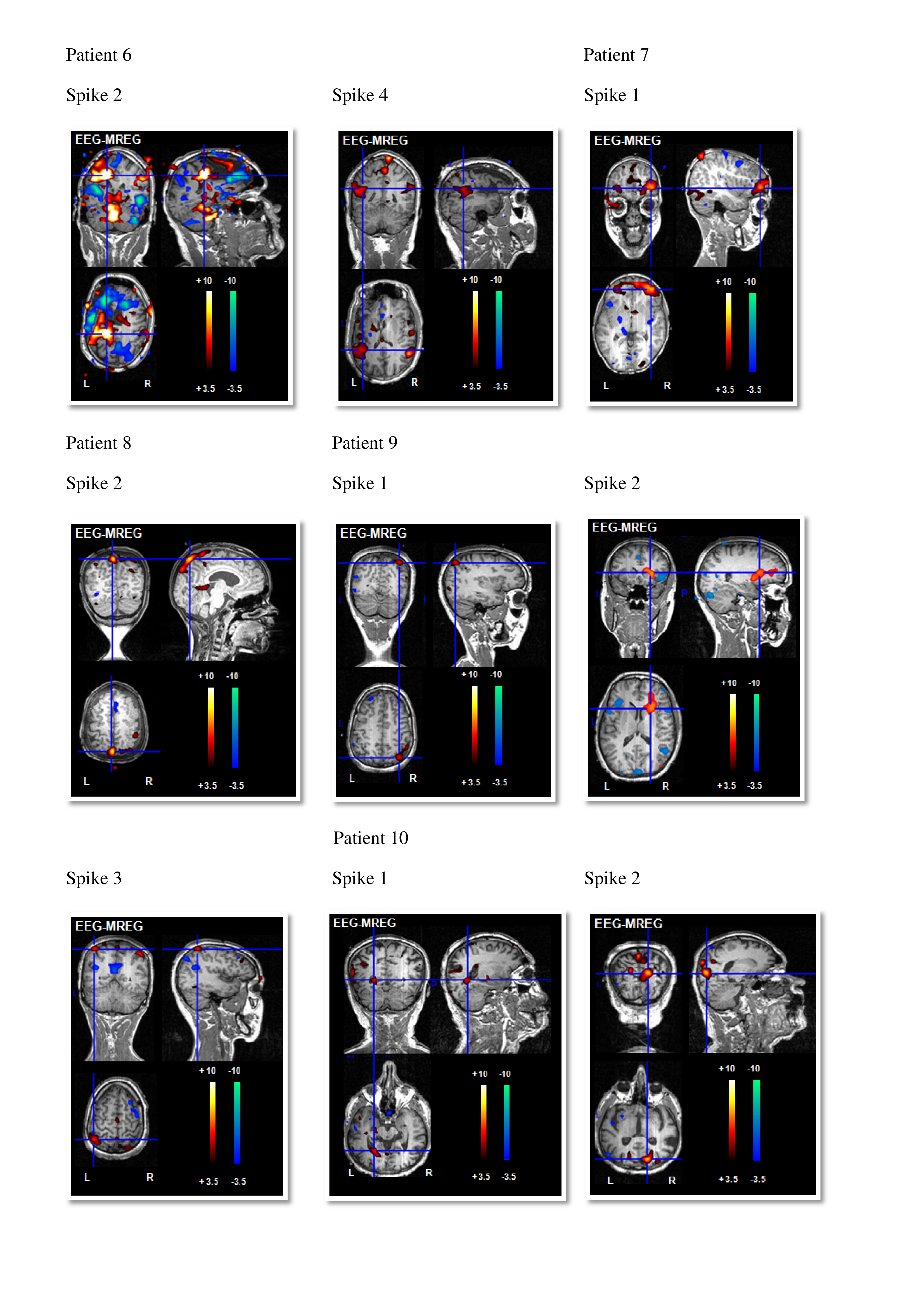

Supplement: S2 Fig — (TIF) [file pone.0140537.s002.tif]

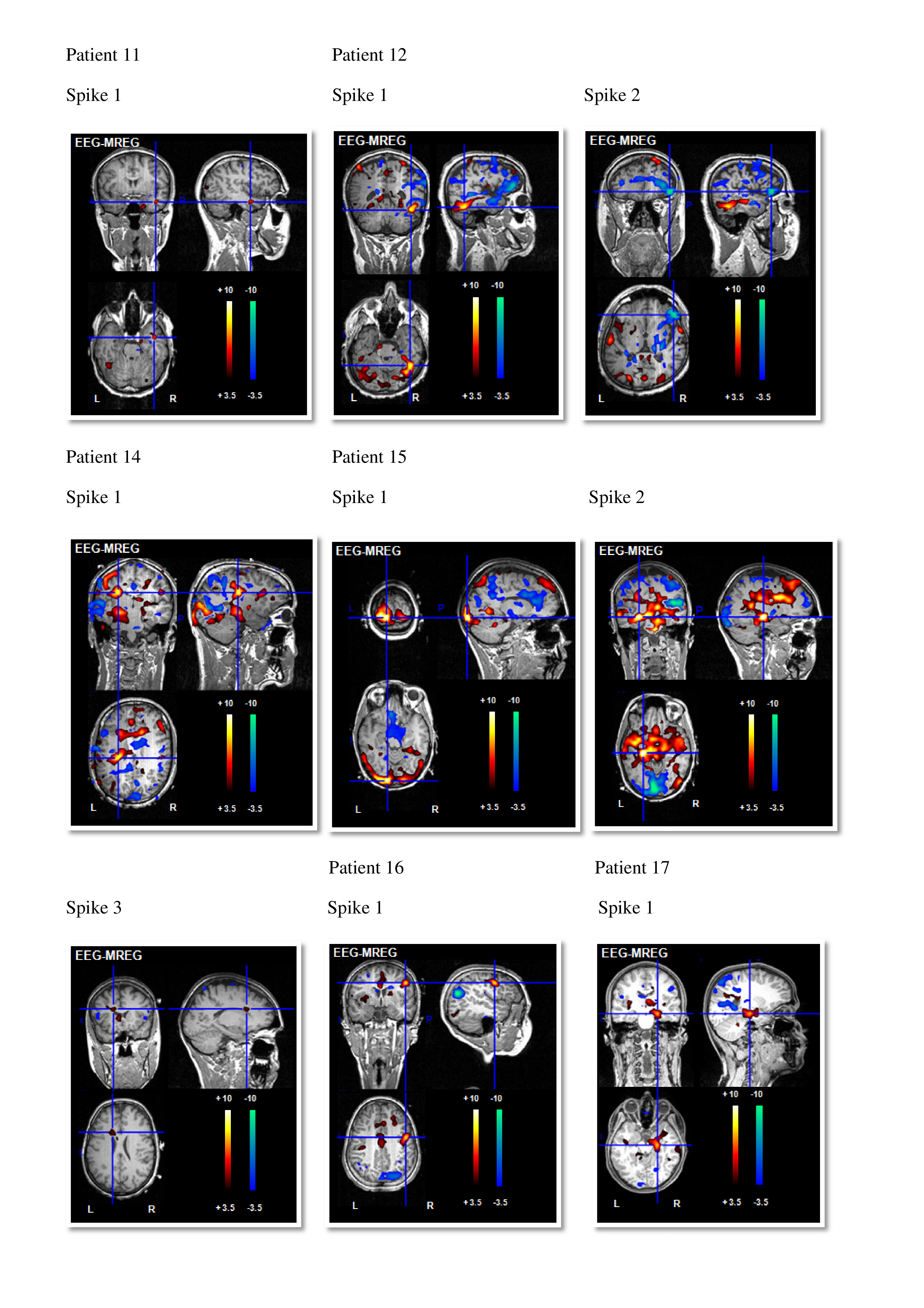

Supplement: S3 Fig — (TIF) [file pone.0140537.s003.tif]

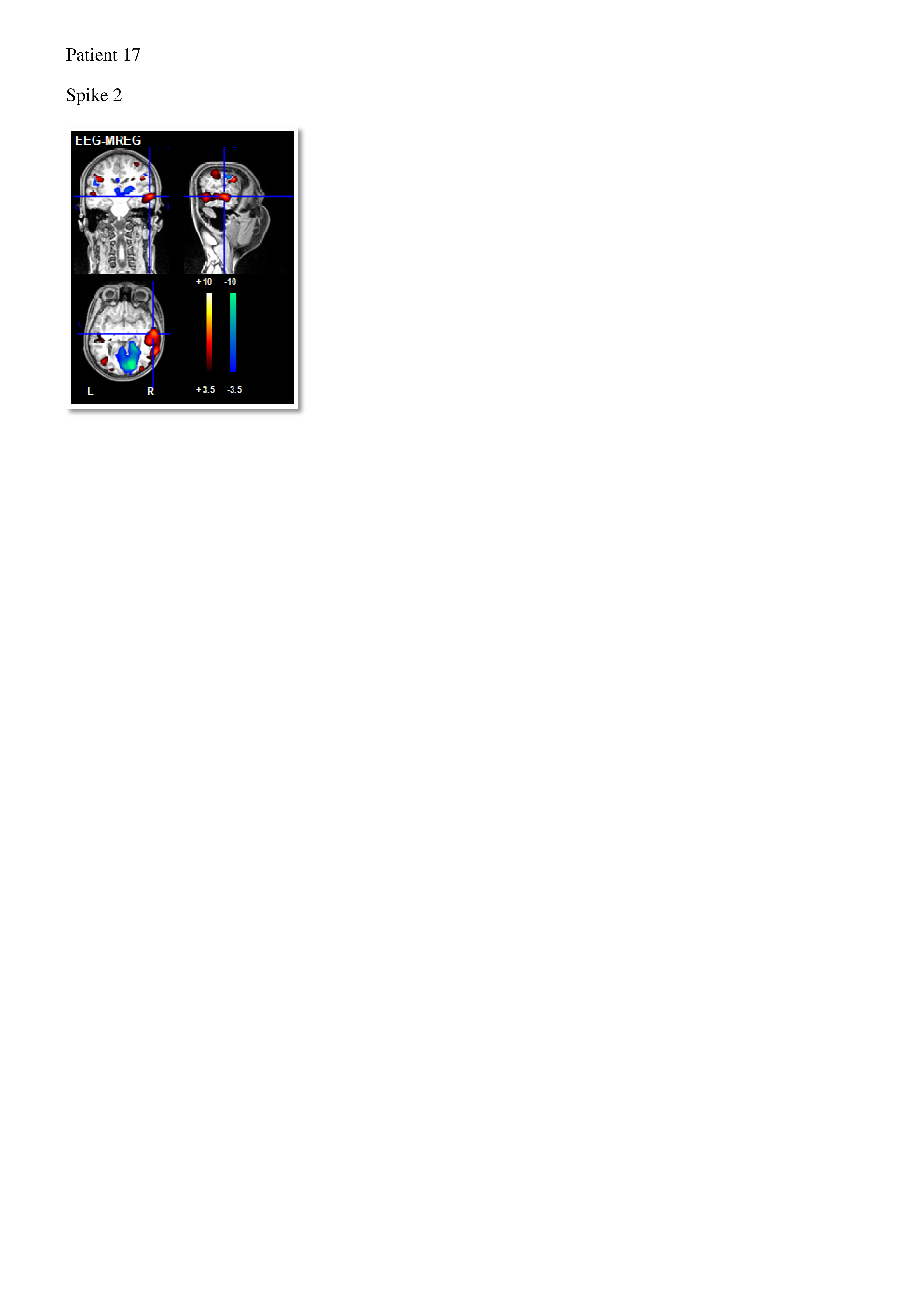

Supplement: S4 Fig — (TIF) [file pone.0140537.s004.tif]
